# Asystole on loop recorder in patients with unexplained syncope and negative tilt testing: age distribution and clinical predictors

**DOI:** 10.1007/s10286-024-01021-8

**Published:** 2024-02-25

**Authors:** Vincenzo Russo, Angelo Comune, Erika Parente, Anna Rago, Andrea Antonio Papa, Gerardo Nigro, Michele Brignole

**Affiliations:** 1 Cardiology and Syncope Unit, Department of Translational Medical Sciences, University of Campania “Luigi Vanvitelli”, Monaldi Hospital, Naples, Italy; 2 Faint & Fall Research Centre, Department of Cardiology, IRCCS Istituto Auxologico Italiano, S. Luca Hospital, Milan, Italy

**Keywords:** Syncope, Asystole, Predictors, Age, Gender, Vasovagal syncope

## Abstract

**Background:**

Approximately 50% of patients with unexplained syncope and negative head-up tilt test (HUTT) who have an electrocardiogram (ECG) documentation of spontaneous syncope during implantable loop recorder (ILR) show an asystolic pause at the time of the event.

**Objective:**

The aim of the study was to evaluate the age distribution and clinical predictors of asystolic syncope detected by ILR in patients with unexplained syncope and negative HUTT.

**Methods:**

This research employed a retrospective, single-center study of consecutive patients. The ILR-documented spontaneous syncope was classified according to the International Study on Syncope of Uncertain Etiology (ISSUE) classification.

**Results:**

Among 113 patients (54.0 ± 19.6 years; 46% male), 49 had an ECG-documented recurrence of syncope during the observation period and 28 of these later (24.8%, corresponding to 57.1% of the patients with a diagnostic event) had a diagnosis of asystolic syncope at ILR: type 1A was present in 24 (85.7%), type 1B in 1 (3.6%), and type 1C in 3 (10.7%) patients. The age distribution of asystolic syncope was bimodal, with a peak at age < 19 years and a second peak at the age of 60–79 years. At Cox multivariable analysis, syncope without prodromes (OR 3.7; *p* = *0.0008*) and use of beta blockers (OR 3.2; *p* = *0.002*) were independently associated to ILR-detected asystole.

**Conclusions:**

In patients with unexplained syncope and negative HUTT, the age distribution of asystolic syncope detected by ILR is bimodal, suggesting a different mechanism responsible for asystole in both younger and older patients. The absence of prodromes and the use of beta blockers are independent predictors of ILR-detected asystole.

**Supplementary Information:**

The online version contains supplementary material available at 10.1007/s10286-024-01021-8.

## Introduction

Head-up tilt test (HUTT) is a useful and necessary diagnostic tool for patients with suspected reflex syncope after initial clinical assessment [1]. Among patients with non-classical vasovagal syncope (VVS), the HUTT positivity rate is 54% [2]. Implantable loop recorder (ILR) is recommended for patients in whom a comprehensive evaluation, including HUTT, failed to identify the cause of transient loss of consciousness [3]. Approximately 50% of patients with unexplained syncope who have spontaneous syncope during ILR monitoring show an asystolic pause at the time of the event [4–6]; however, no data are still available regarding the clinical characteristics of this subgroup. The early identification of patients with unexplained syncope at high probability to develop asystole might be useful for fast-track access to ILR. The aim of this study was to investigate the age distribution and the clinical predictors of asystole during ILR monitoring.

## Methods

We retrospectively evaluated all consecutive patients with unexplained syncope and negative HUTT who had undergone ILR implantation at the Syncope Unit of the University of Campania “Luigi Vanvitelli” – Monaldi Hospital of Naples, Italy from 1 March 2019 to 1 May 2022. The diagnostic workup, which included electrocardiogram (ECG), echocardiogram, 24-h Holter ECG monitoring, standing test, carotid sinus massage, and HUTT, was inconclusive for all included patients. Other diagnostic tests were performed in selected cases when deemed necessary. Patients with suspected cardiac syncope were carefully excluded. Specifically, exclusion criteria were: (i) suspected cardiac arrhythmic syncope [inadequate sinus bradycardia (< 50 beats per min, bpm) or sinoatrial block, second-degree Mobitz I atrioventricular block, second-degree Mobitz II or third-degree atrioventricular block, paroxysmal tachyarrhythmia or ventricular tachycardia, and bundle branch block]; (ii) severe structural heart disease and/or significant ECG abnormalities; (iii) orthostatic hypotension; and (iv) non-syncopal causes of transient loss of consciousness. The patients who had the typical features of reflex syncope or a positive HUTT response were excluded. In these patients the diagnosis of reflex syncope was considered established even if not confirmed by tests.

ILRs (Biomonitor IIIM, Biotronik, Berlin, Germany) were implanted with standard techniques and programmed to detect atrial fibrillation with > 12% R-R interval variability, bradycardia episodes with < 40 bpm heart rate, high ventricular heart rate episodes with > 180 bpm, sudden rate drops > 40%, and asystole episodes > 3 s. The ILR-documented spontaneous syncope was classified according to the ISSUE classification [6]. Asystole was defined as a syncope occurring in the presence of sinus arrest (type 1A), sinus bradycardia plus atrioventricular (AV) block (type 1B), or sudden AV block (type 1C) and R-R interval ≥ 3 s. The prevalence and clinical predictors of ILR-detected asystole were assessed. This study was conducted according to the Declaration of Helsinki and approved by the institutional ethics committees (ID-168/02032021); a written informed consent for data collection was obtained from the patients.

### Statistical analysis

Categorical data were expressed as number and percentage and were compared by Fisher’s exact test. Continuous variables were expressed as either median [interquartile range (IQR)] or mean ± standard deviation (SD) on the basis of their distribution (normal or not) as assessed by the Kolmogorov–Smirnov and the Shapiro–Wilk tests. Parametric Student’s *t*-test was used to compare continuous variable. Univariable and multivariable (stepwise model) Cox regression analyses were performed to evaluate the individual and independent association of clinical variables with the occurrence of ILR-detected asystole and presented as odds ratio (OR) with their 95% confidence intervals (CI). Multicollinearity was assessed using collinearity diagnostics, and the covariates with variance inflation factors > 2.5 were excluded from the regression analysis. A two-sided probability *p*-value < 0.05 was considered statistically significant. All analyses were performed using SPSS statistical software version 24.0 (SPSS, Chicago, Illinois, USA) and STATA 14.0 software (StataCorp, College Station, Texas, USA).

## Results

A total of 113 patients with ILR (54 ± 19.6 years; 46% male) were included in the study and were followed for a median follow-up of 22 (IQR 9–53) months. Of these, 49 patients (43.3%) had an ECG documentation of syncope recurrence (Table 1): 28 patients (24.8%, corresponding to 57.1% of the patients with a diagnostic event) had a diagnosis of asystolic syncope at ILR [type 1A was present in 24 (85.7%), type 1B in 1 (3.6%), and type 1C in 3 (10.7%) patients] according to the ISSUE classification; another 21 patients (18.6%, corresponding to 42.9% of patients with a diagnostic event) had non-asystolic syncope (classes 2, 3, and 4 of the ISSUE classification). The patients with asystolic syncope were compared with the pooled data of patients with non-asystolic syncope and those without syncope, because the two subgroups had similar clinical features (Supplementary Table 1).

**Table 1:** Baseline characteristics of study population

	Overall population *(n. 113)*	Asystole group *(n. 28)*	Non-asystole group *(n. 85)*	*p*-value
Age (years), mean ± SD	54.0 ± 19.6	60.7 ± 18.2	51.8 ± 19.7	0.04
Male gender, *n* (%)	52 (46)	15 (53.6)	37 (43.5)	0.36
Smoking, *n* (%)	35 (30.9)	8 (28.6)	27 (31.8)	0.75
Hypertension, *n* (%)	50 (44.2)	19 (67.9)	31 (36.5)	0.003
Diabetes mellitus, *n* (%)	14 (12.4)	6 (22.2)	8 (9.4)	0.08
CAD, *n* (%)	13 (11.5)	7 (25)	6 (7)	0.01
HCM, *n* (%)	4 (3.5)	1 (3.6)	3 (3.5)	0.99
DCM, *n* (%)	3 (2.6)	2 (7.1)	1 (1.1)	0.09
AF, *n* (%)	8 (7)	3 (10.7)	5 (5.9)	0.39
RBBB, *n* (%)	8 (7)	1 (3.6)	7 (8.2)	0.40
Syncope without prodromes, *n* (%)	31 (27.4)	16 (57.1)	15 (17.6)	< 0.0001
Traumatic syncope, *n* (%)	42 (37.1)	12 (42.9)	30 (35.3)	0.48
Syncope during sitting/supine position, *n* (%)	27 (23.9)	5 (17.9)	22 (25.9)	0.39
Driving syncope, *n* (%)	7 (6.2)	1 (3.5)	6 (7)	0.57
Supine SBP values (mmHg), mean ± SD	130.3 ± 19.6	135.2 ± 15.8	128.5 ± 20.8	0.12
Supine DBP values (mmHg), mean ± SD	78.8 ± 12.7	78.3 ± 12.8	79 ± 12.8	0.8
Supine heart rate (bpm), mean ± SD	69.8 ± 13.7	69.5 ± 14.8	69.8 ± 13.5	0.9
Alpha blockers, *n* (%)	2 (1.8)	1 (3.5)	1 (1.2)	0.4
Beta blockers, *n* (%)	29 (25.7)	15 (53.6)	14 (16.5)	0.0001
Calcium channel antagonists, *n* (%)	14 (12.4)	5 (17.9)	9 (10.6)	0.31
ACE-i/ARBs, *n* (%)	37 (32.7)	14 (50)	23 (27.1)	0.025
Diuretics, *n* (%)	23 (20.3)	10 (35.7)	13 (15.3)	0.01
Insulin, *n* (%)	5 (4.4)	1 (3.5)	4 (4.7)	0.79
Oral hypoglycemics, *n* (%)	11 (9.7)	5 (17.9)	6 (7)	0.09

*CAD* coronary artery disease, *HCM* hypertrophic cardiomyopathy, *DCM* dilated cardiomyopathy, *AF* atrial fibrillation, *RBBB* right bundle branch block, *SBP* systolic blood pressure, *DBP* diastolic blood pressure, *ACE-I* angiotensin converting enzyme inhibitors, *ARB* angiotensin II receptor blockers, *CKD* chronic kidney disease

**Fig. 1:**
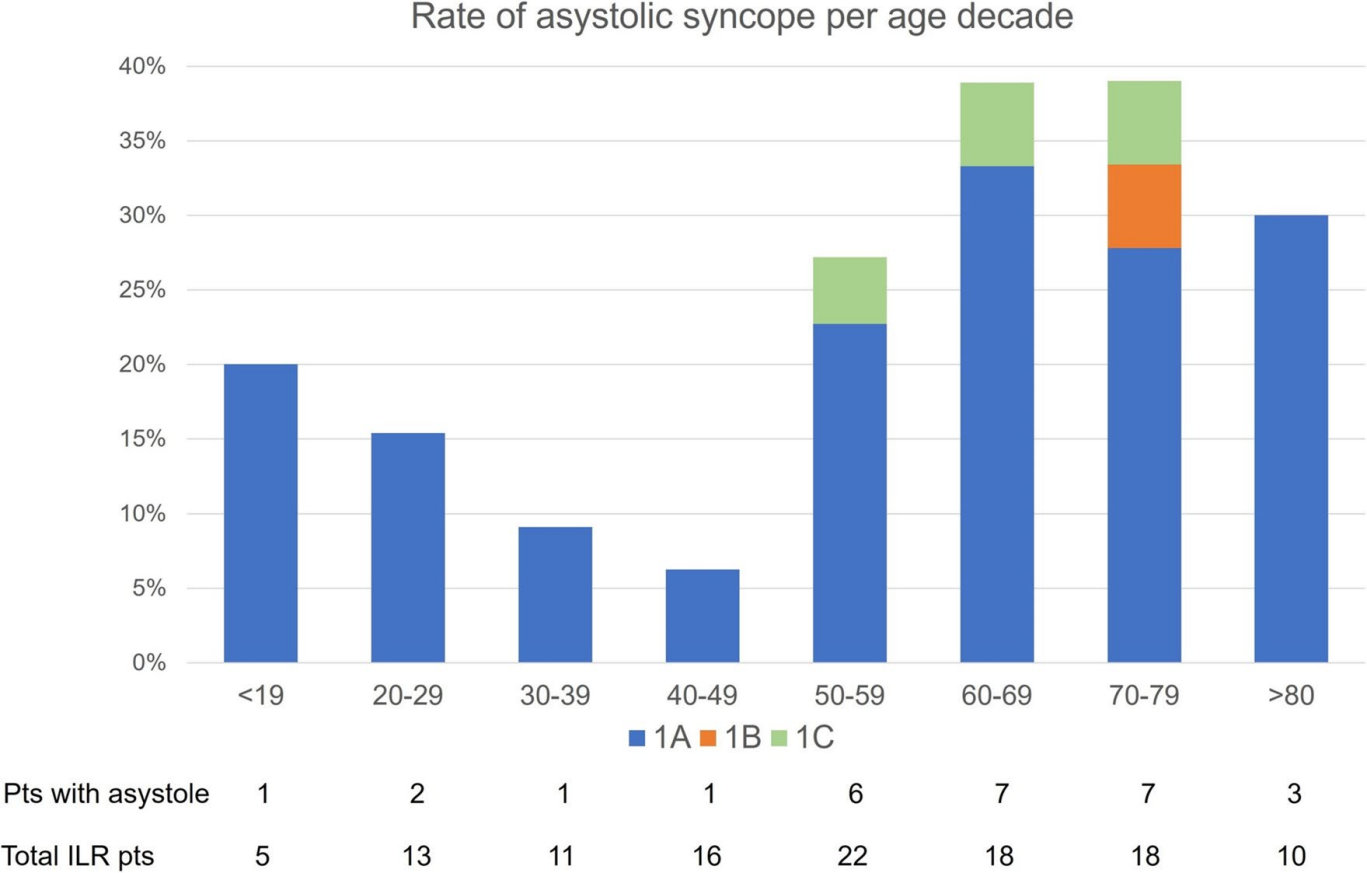
Age distribution (per decades) of ILR detected asystole during lifespan

**Fig. 2:**
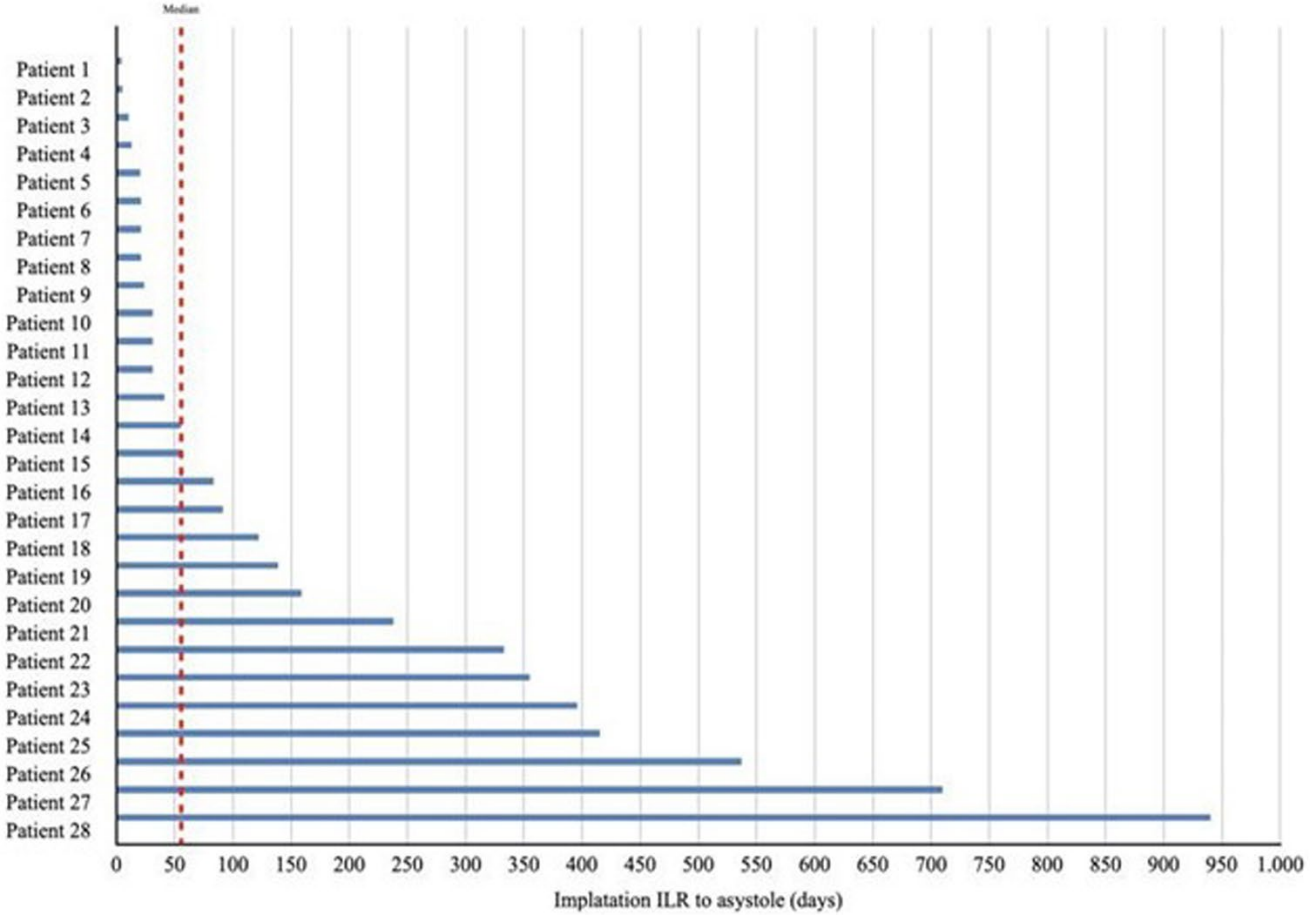
Time from ILR implantation to asystole detection

The age distribution of asystolic syncope showed a bimodal distribution with two peaks at 0–19 years and 60–79 years (Fig. 1). Types 1B and 1C were only detected among patients older than 50 years. The time from ILR implantation to asystole detection was shown in Fig. 2.

Older age, hypertension, coronary artery disease, history of syncope without prodromes, and use of beta blockers, ACE-i/ARBs, and diuretic drugs were more frequent in patients with ILR detected asystole than in the others. No patients aged ≤ 30 years were taking any drugs. At multivariate Cox regression analysis, syncope without prodromes (OR 3.7; *p* = *0.0008*) and use of beta blockers (OR 3.2; *p* = *0.002*) were independently associated with ILR-detected asystole (Table 2).

**Table 2:** Univariable and multivariable Cox regression analysis for clinical characteristics associated with ILR-detected asystole

	Univariable analysis		Multivariable analysis	
	OR (95% CI)	*p*	OR (95% CI)	*p*
Age (every 10 years)	1.02 (1.00–1.05)	0.04	–	–
Male gender	1.5 (0.6–3.5)	0.36	–	–
Smoking	0.86 (0.33–2.2)	0.75	–	–
Hypertension	3.7 (1.5–9.1)	0.003	–	–
Diabetes mellitus	2.7 (0.8–8.8)	0.09	–	–
CAD	4.4 (1.3–14.4)	0.015	–	–
HCM	1.1 (0.1–11.1)	0.9	–	–
DCM	6.5 (0.6–74.2)	0.1	–	–
AF	1.9 (0.42–8.6)	0.4	–	–
RBBB	0.41 (0.05–3.5)	0.41	–	–
Syncope without prodromes	6.2 (2.4–15.8)	0.0001	3.7 (1.7–7.9)	0.0008
Traumatic syncope	1.4 (0.57–3.3)	0.47	–	–
Syncope during sitting/supine position	0.62 (0.2–1.8)	0.39	–	–
Driving syncope	0.5 (0.06–4.2)	0.5	–	–
Supine SBP values	1.01 (0.99–1.04)	0.14	–	–
Supine DBP values	0.99 (0.96–1.03)	0.8	–	–
Supine heart rate	0.99 (0.96–1.03)	0.9	–	–
Alpha blockers	3.1 (0.2–51.4)	0.4	–	–
Beta blockers	5.8 (2.3–14.9)	0.0002	3.2 (1.5–6.9)	0.002
Calcium channel antagonists	1.8 (0.5–6.0)	0.3	–	–
ACE-i/ARBs	2.7 (1.1–6.5)	0.02	–	–
Diuretics	3.07 (1.2– 8.1)	0.02	–	–
Insulin	0.74 (0.07–6.9)	0.79	–	–
Oral hypoglycemics	2.7 (0.8–10.3)	0.1	–	–

*CAD* coronary artery disease, *HCM* hypertrophic cardiomyopathy, *DCM* dilated cardiomyopathy, *AF* atrial fibrillation, *RBBB* right bundle branch block, *SBP* systolic blood pressure, *DBP* diastolic blood pressure, *ACE-I* angiotensin converting enzyme inhibitors, *ARB* angiotensin II receptor blockers, *CKD* chronic kidney disease

## Discussion

The main findings of the present study can be summarized as follows: asystolic syncope accounted for 24.8% of patients with unexplained syncope and negative HUTT who had received an ILR monitoring; this figure corresponds to 57.1% of those with a diagnostic event. An age-related bimodal distribution of ILR-detected asystole, characterized by two peaks at < 19 years and at 60–79 years of age, was observed. The history of syncope without prodromes and the use of beta blockers were the only independent predictors of ILR-detected asystole leading to syncope. Our results support a strategy based on early diagnostic ILR application in patients with unexplained syncope without prodromes or during beta blocker therapy.

The relationship of asystolic syncope between ILR and HUTT has already been assessed in the literature [4–6]. In the ISSUE 1 study [5], asystolic syncope was diagnosed in 11 (46%) out of 24 patients affected by isolated syncope who had an ECG documentation of the event; another 13 patients (54%) had a diagnosis of non-asystolic syncope. The prevalence and type of asystolic syncope were not different from the 62% prevalence observed in HUTT-positive patients who had an ECG documentation of the event, suggesting a unique pathophysiology in the two populations and a low sensitivity of HUTT in predicting spontaneous asystolic syncope. Similarly, in the ISSUE 2 study [6], asystolic syncope was diagnosed in 30 (54%) out of 56 patients with negative HUTT who had an ECG documentation of the event and in 17 (45%) out of 38 patients with positive HUTT. In a meta-analysis [4] of 49 studies, including 4381 subjects with unexplained syncope, ILR findings were diagnostic in 43.9% of cases; a bradyarrhythmia requiring a permanent pacemaker implantation was present in 18.2%, which corresponds to approximately 41% of diagnostic tests. The results of the present study are consistent with the above data.

Among our study population, no association between ILR-detected asystole leading to syncope and age or gender was shown. This finding is consistent with our previous evidence in patients with HUTT-induced asystole and confirm the hypothesis of the lack of a significant sex and gender effect on the prevalence of spontaneous asystole [7]. Nevertheless, an original finding of the present study was the age-related bimodal distribution of asystolic syncope, with types 1B and 1C limited to patients aged > 70 years, suggesting a different mechanism of asystole between younger and older patients. Our results are consistent with the study of Torabi et al. [8], who found a similar bimodal age distribution of the first syncopal episode in a large population of 1928 patients. One hypothesis suggests that unexplained syncope starting at advanced age is an expression of a complex pathological (degenerative) process of the autonomic function system [9], whereas unexplained syncope starting at a young age is an isolated para-physiological phenomenon that could be the expression of an excessive defense mechanism against situations of hypersympathetic tone, such as during prolonged orthostatic challenge with hypotensive tendency or emotional stress [10]. In some older patients, the vagal burst may be the consequence of a pathological process, unrelated to the physiological neural control of usual conditions (e.g., standing-up, physical exercise, etc.). In addition, the magnitude of the final effect on cardiac effectors is likely to be enhanced by an intrinsic sinus node dysfunction and decrease of AV node conduction properties, as suggested by types 1B and 1C forming in older patients only, and by the decreased sympathetic drive in older individuals.

History of syncope without prodromes is a strong independent predictor of ILR-detected asystole leading to syncope. In the clinical context of vasovagal syncope, the prodromes are related to hypotensive mechanism and may contribute to identifying patients with the so-called hypotensive phenotype of reflex syncope [11]; in contrast, their absence suggests a rapid onset asystole as main determinant of syncope. This clinical feature may be used to identify patients with unexplained syncope in need of fast-track access to ILR implantation. The early detection of underlying mechanisms may lead to the application of a specific treatment to prevent both recurrences and associated physical injuries.

No previous studies have evaluated the association between pharmacological therapy and ILR-detected arrhythmias among patients with unexplained syncope. Our data suggest a positive association between the use of beta blockers and ILR-detected asystole. Since the recurrence of syncope can be reduced by discontinuing/reducing vasoactive therapy in most elderly patients affected by syncope [12], we can speculate that the discontinuation of beta blockers may favorably impact the recurrence of syncope with asystole.

## Limitations

Our results should be interpreted considering the limitations related to the study’s retrospective observational single-center nature. ILR is unable to provide a correlation between electrocardiographic findings and arterial blood pressure or cerebral blood flow; indeed, the underlying mechanism (i.e., intrinsic cardiac versus extrinsic reflex) may remain uncertain and the exact etiology can only be inferred. The proposed strategy of early diagnostic ILR application in patients with unexplained syncope without prodromes or during beta blocker therapy might be hampered by several confounding factors, e.g., selection biases and filters at different ages that limit the external validity of the study. The clinical variable "syncope without prodromes" might be biased by amnesia from loss of consciousness, which is common in the elderly [13].

## Conclusions

In patients with unexplained syncope and negative HUTT, the age distribution of asystolic syncope detected by ILR is bimodal, suggesting a different mechanism responsible for asystole in both younger and older patients. The absence of prodromes and the use of beta blockers are independent predictors of ILR-detected asystole. These data might be useful to identify patients in need of fast-track access to ILR implantation for the early detection of asystole in need of specific treatment.

### Supplementary Information

Below is the link to the electronic supplementary material.

Supplementary file1 (DOCX 20 KB)

## Data Availability

The data that support the findings of this study are available from the corresponding author, upon reasonable request.

## References

[1] Sutton R, Fedorowski A, Olshansky B (2021). Tilt testing remains a valuable asset. Eur Heart J.

[2] Russo V, Parente E, Comune A (2023). The clinical presentation of syncope influences the head-up tilt test responses. Eur J Intern Med.

[3] Brignole M, Moya A, de Lange FJ, ESC Scientific Document Group (2018). 2018 ESC Guidelines for the diagnosis and management of syncope. Eur Heart J.

[4] Solbiati M, Casazza G, Dipaola F (2017). The diagnostic yield of implantable loop recorders in unexplained syncope: a systematic review and meta-analysis. Int J Cardiol.

[5] Moya A, Brignole M, Menozzi C (2001). International Study on Syncope of Uncertain Etiology (ISSUE) Investigators Mechanism of syncope in patients with isolated syncope and in patients with tilt-positive syncope. Circulation.

[6] Brignole M, Sutton R, Menozzi C (2006). International Study on Syncope of Uncertain Etiology 2 (ISSUE 2) Group. Lack of correlation between the responses to tilt testing and adenosine triphosphate test and the mechanism of spontaneous neurally mediated syncope. Eur Heart J.

[7] Russo V, Parente E, Rago A (2022). Cardioinhibitory syncope with asystole during nitroglycerin potentiated head up tilt test: prevalence and clinical predictors. Clin Auton Res.

[8] Torabi P, Rivasi G, Hamrefors V (2022). Early and late-onset syncope: insight into mechanisms. Eur Heart J.

[9] Alboni P, Brignole M, Degli Uberti EC (2007). Is vasovagal syncope a disease?. Europace.

[10] Alboni P, Alboni M (2017). Typical vasovagal syncope as a “defense mechanism” for the heart by contrasting sympathetic overactivity. Clin Auton Res.

[11] Brignole M, Rivasi G, Sutton R (2021). Low-blood pressure phenotype underpins the tendency to reflex syncope. J Hypertens.

[12] Solari D, Tesi F, Unterhuber M (2017). Stop vasodepressor drugs in reflex syncope: a randomised controlled trial. Heart.

[13] O'Dwyer C, Bennett K, Langan Y, Fan CW, Kenny RA (2011). Amnesia for loss of consciousness is common in vasovagal syncope. Europace.

