# Supplementary material for: Asystole on loop recorder in patients with unexplained syncope and negative tilt testing: age distribution and clinical predictors
Source: Clin Auton Res. 2024 Feb 25;34(1):137–42. doi: 10.1007/s10286-024-01021-8 (PMC10944445; doi:10.1007/s10286-024-01021-8)
Supplement: Supplementary file 1 — Supplementary file1 (DOCX 20 KB) [file 10286_2024_1021_MOESM1_ESM.docx]

**Supplementary Table 1:** Baseline characteristics of ILR patients with non-asystole syncope (No asystole syncope Group) compared to those without syncope (No-Syncope Group)

|  | **No Asystole Syncope Group**  *(n. 21)* | **No Syncope Group**  *(n. 64)* | ***P-value*** |
| --- | --- | --- | --- |
| Age (years), mean ± SD | 50.7 ± 21.8 | 52.1 ± 19.1 | 0.77 |
| Male gender, n (%) | 10 (47.6) | 27 (42.2) | 0.66 |
| Smoking, n (%) | 4 (19) | 23 (35.9) | 0.15 |
| Hypertension, n (%) | 8 (38.1) | 23 (35.9) | 0.86 |
| Diabetes mellitus, n (%) | 1 (4.8) | 7 (10.9) | 0.4 |
| CAD, n (%) | 3 (14.3) | 3 (4.6) | 0.14 |
| HCM, n (%) | 0 (0) | 3 (4.6) | 0.31 |
| DCM, n (%) | 1 (4.7) | 0 (0) | 0.08 |
| AF, n (%) | 2 (9.5) | 3 (4.7) | 0.42 |
| RBBB, n (%) | 2 (9.5) | 5 (7.8) | 0.8 |
| Syncope without prodroms, n (%) | 4 (19) | 11 (17.2) | 0.84 |
| Traumatic syncope, n (%) | 8 (38.1) | 22 (34.4) | 0.76 |
| Syncope during sitting/supine position, n (%) | 5 (23.8) | 17 (26.6) | 0.8 |
| Driving Syncope, n (%) | 1 (4.8) | 5 (7.8) | 0.64 |
| Supine SBP values (mmHg), mean ± SD | 128.7± 23.9 | 128.5 ± 19.8 | 0.95 |
| Supine DBP values (mmHg), mean ± SD | 74.2 ± 8.8 | 80.6± 13.6 | 0.05 |
| Supine Heart Rate (bpm), mean ± SD | 66.9 ± 10.8 | 70.8± 14.1 | 0.9 |
| Alfa-Blockers, n (%) | 1 (4.8) | 0 (0) | 0.08 |
| Beta-Blockers, n (%) | 5 (23.8) | 9 (14.1) | 0.3 |
| Calcium Channel Antagonists, n (%) | 3 (14.3) | 6 (9.4) | 0.53 |
| ACE-i/ARBs, n (%) | 6 (28.6) | 17 (26.6) | 0.86 |
| Diuretics, n (%) | 4 (19) | 9 (14.1) | 0.6 |
| Insulin, n (%) | 0 | 4 (6.2) | 0.24 |
| Oral Hypoglicemics, n (%) | 1 (4.8) | 5 (7.8) | 0.64 |

Abbreviations: CAD: coronary artery disease; HCM: hypertrophic cardiomyopathy; DCM: dilated cardiomyopathy; AF: atrial fibrillation; RBBB: right bundle branch block; SBP: systolic blood pressure; DBP: diastolic blood pressure; ACE-I: angiotensin converting enzyme inhibitors; ARB: Angiotensin II receptor blockers; CKD: chronic kidney disease.
